# A New Search Method Using Association Rule Mining for Drug-Drug Interaction Based on Spontaneous Report System

**DOI:** 10.3389/fphar.2018.00197

**Published:** 2018-03-09

**Authors:** Yoshihiro Noguchi, Anri Ueno, Manami Otsubo, Hayato Katsuno, Ikuto Sugita, Yuta Kanematsu, Aki Yoshida, Hiroki Esaki, Tomoya Tachi, Hitomi Teramachi

**Affiliations:** Laboratory of Clinical Pharmacy, Gifu Pharmaceutical University, Gifu, Japan

**Keywords:** drug-drug interaction, spontaneous reporting systems, signal detection, association rule mining, apriori algorithm

## Abstract

**Background:** Adverse events (AEs) can be caused not only by one drug but also by the interaction between two or more drugs. Therefore, clarifying whether an AE is due to a specific suspect drug or drug-drug interaction (DDI) is useful information for proper use of drugs. Whereas previous reports on the search for drug-induced AEs with signal detection using spontaneous reporting systems (SRSs) are numerous, reports on drug interactions are limited. This is because in methods that use “a safety signal indicator” (signal), which is frequently used in pharmacovigilance, a huge number of combinations must be prepared when signal detection is performed, and each risk index must be calculated, which makes interaction search appear unrealistic.

**Objective:** In this paper, we propose association rule mining (AR) using large dataset analysis as an alternative to the conventional methods (additive interaction model (AI) and multiplicative interaction model (MI)).

**Methods:** The data source used was the Japanese Adverse Drug Event Report database. The combination of drugs for which the risk index is detected by the “combination risk ratio (CR)” as the target was assumed to be true data, and the accuracy of signal detection using the AR methods was evaluated in terms of sensitivity, specificity, Youden's index, *F*-score.

**Results:** Our experimental results targeting Stevens-Johnson syndrome indicate that AR has a sensitivity of 99.05%, specificity of 92.60%, Youden's index of 0.917, *F*-score of 0.876, AI has a sensitivity of 95.62%, specificity of 96.92%, Youden's index of 0.925, and *F*-score of 0.924, and MI has a sensitivity of 65.46%, specificity of 98.78%, Youden's index of 0.642, and *F*-score of 0.771. This result was about the same level as or higher than the conventional method.

**Conclusions:** If you use similar calculation methods to create combinations from the database, not only for SJS, but for all AEs, the number of combinations would be so enormous that it would be difficult to perform the calculations. However, in the AR method, the “Apriori algorithm” is used to reduce the number of calculations. Thus, the proposed method has the same detection power as the conventional methods, with the significant advantage that its calculation process is simple.

## Introduction

Pharmacovigilance is defined as “the science and activities relating to the detection, assessment, understanding and prevention of drug-related problems” by the World Health Organization (WHO) (World Health Organization, 2002). For adverse drug events (ADEs) that cannot be found in clinical trials, numerous risk assessments of drugs using spontaneous reporting systems (SRSs) based on spontaneous reports of drug-adverse event (AE) pairs including post-marketing survey data have been reported (Poluzzi et al., 2013; Zorych et al., 2013; Fujimoto et al., 2014; Ali et al., 2015; Noguchi et al., 2015; Mizuno et al., 2016; Gahr et al., 2017).

For risk assessment, the data mining algorithms of the quantitative signal detection from such a large database, include the proportional reporting ratio (PRR) (Evans et al., 2001) is used in the Medicines and Healthcare Products Regulatory Agency (MHRA), the reporting odds ratio (ROR) (van Puijenbroek et al., 2002) which is used by the Netherlands Pharmacovigilance Centre Lareb, the information component (IC) as Bayesian Confidence Propagation Neural Network (BCPNN) (Bate et al., 1998) is used by the Uppsala Monitoring Centre, Sweden, and the Gamma-Poisson Shrinker (GPS) (Szarfman et al., 2002) as empirical Bayes geometric mean (EBGM). These methods are useful for early detection of unknown ADEs. However, in pre-marketing randomized clinical trials, patients with multiple drug use are usually excluded because focus is on establishing the safety and efficacy of single drugs and not on the investigation of drug-drug interactions (DDIs).

However, the reality is that in post-marketing pharmaceutical products, unlike pre-marketing trials, not only ADEs caused by a single drug but also ADEs caused by DDI by two or more drugs exist. The proportion of ADEs due to DDI is estimated to be up to 30% of unexpected ADEs (Pirmohamed and Orme, 1998). Therefore, clarifying whether the ADEs expressed are due to a specific suspected drug or DDI would be useful when a patient uses the drugs.

To take into consideration the interaction between Drug A and Drug B, as in the case of a single drug, a “Drug A and Drug B-AE 2 × 2 contingency table” should first be prepared and the respective signal values calculated. Furthermore, to detect the signal at the time of concomitant use as a DDI, it must be stronger than the signal of each single drug.

Methods such as the additive interaction model (AI) (= risk difference) and the multiplicative interaction model (MI) (= risk ratio) are used to evaluate interactions with ADEs expression when two drugs are used in combination (Thakrar et al., 2007). However, in searching for drug interactions, Susuta et al. proposed “combination risk ratio (CR),” in which the ratio of signal detection using a plurality of drugs in combination to the signal detection calculated by focusing on one drug is utilized (Susuta and Takahashi, 2014). If the PRR value of ADE when two concomitant drugs are used is more than twice the PRR value of each drug, then it is regarded as a DDI. This method proposed by Susuta et al. is used searching for drug interactions in Japan (Noguchi et al., 2015; Mizuno et al., 2016).

However, in these methods as AI, MI, and CR, it is necessary to compose a huge combination of medicines from SRS and compare the signal of these drugs with the signal of a single drug in order to detect drug interaction. Consequently, it is difficult to efficiently conduct a cross-sectional search aimed at early detection of DDI.

Association rule mining (AR) is used to find “interesting patterns hidden in a database.” Algorithms that simplify operations such as the “Apriori algorithm” (Agrawal and Srikant, 1994) are used for AR and, in recent years, utilization of ADEs as signal detection has also been reported (Shirakuni et al., 2009; Harpaz et al., 2010; Wang et al., 2012; Fujiwara et al., 2016). Therefore, to construct a simple ADE search method for DDI, we modified existing AR such that risk variation due to a combination of drugs can be evaluated and investigated combinations of drugs that may cause Stevens-Johnson syndrome (SJS) as DDI.

## Materials and methods

### Data sources

**Figure 1:**
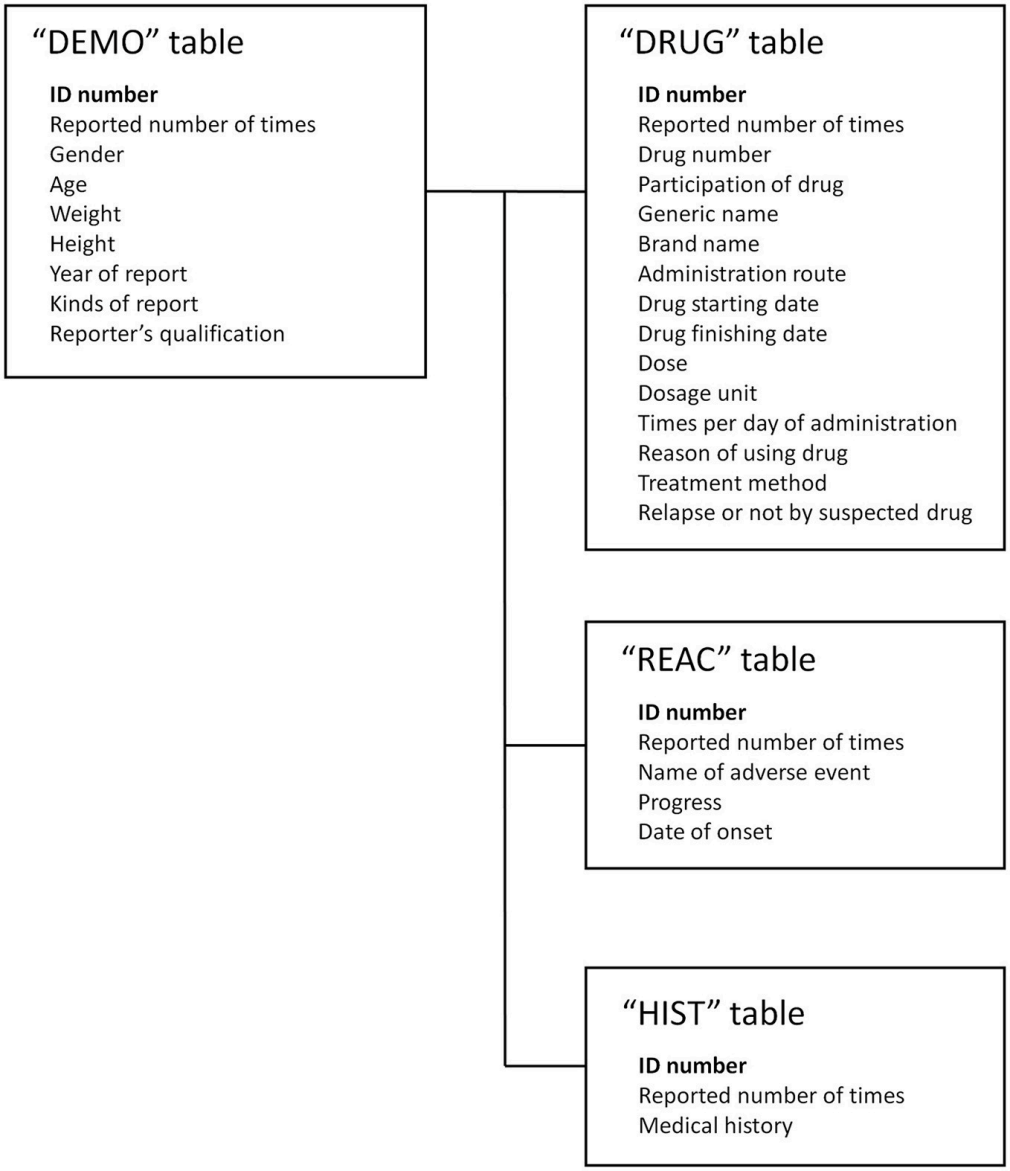
Four information tables included in Japanese Adverse Drug Event Report.

We used the SRS dataset from the first quarter of 2004 to the fourth quarter of 2015 from the Japanese Adverse Drug Event Report (JADER) database. The JADER database was downloaded from the website of the Pharmaceuticals and Medical Devices Agency in Japan. The structure of JADER complies with the international safety reporting guidelines (ICH E2B). The database consists of four tables: “DEMO,” “DRUG,” “REAC,” and “HIST.” The “DEMO” table contains information on gender, age, weight, etc., The “DRUG” table contains information on suspect drug, concomitant usage, etc. The “REAC” table contains information on AE and outcome. The “HIST” table contains information on primary disease, secondary disease, etc. In this study, we combined these four tables (Figure 1) and analyzed 374,327 cases, with cases having gender and age deficiency and cases that have ambiguous reports of age such as “youth” and “elderly” being excluded.

### Definitions of adverse drug events

The drugs targeted for the survey are all registered and classified as “suspect drugs” in JADER. The AE investigated was SJS. To extract the AEs from JADER, the preferred term (PT) in the Medical Dictionary for Regulatory Activities (Japanese version (MedDRA /J) version 18.1) was used. Further, the definition of SJS was the same as that used by Susuta et al. in a previous study (Susuta and Takahashi, 2014).

### Association rule mining model

**Figure 2:**
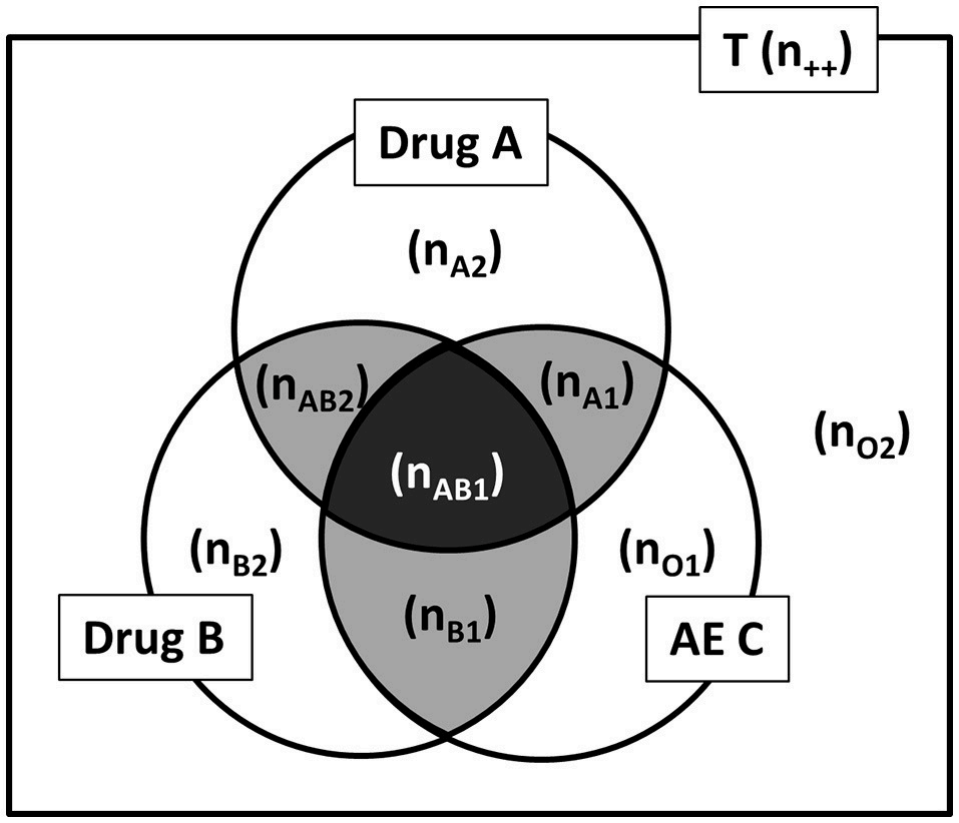
Drug A, Drug B, and AE C by Venn diagram from the spontaneous report system. AE, adverse events; T, total of report (all reports); n, the number of reports (e.g., n_++_, the number of all reports; n_AB1_, the number of Drug A and Drug B induced AE C report).

**Figure 3:**
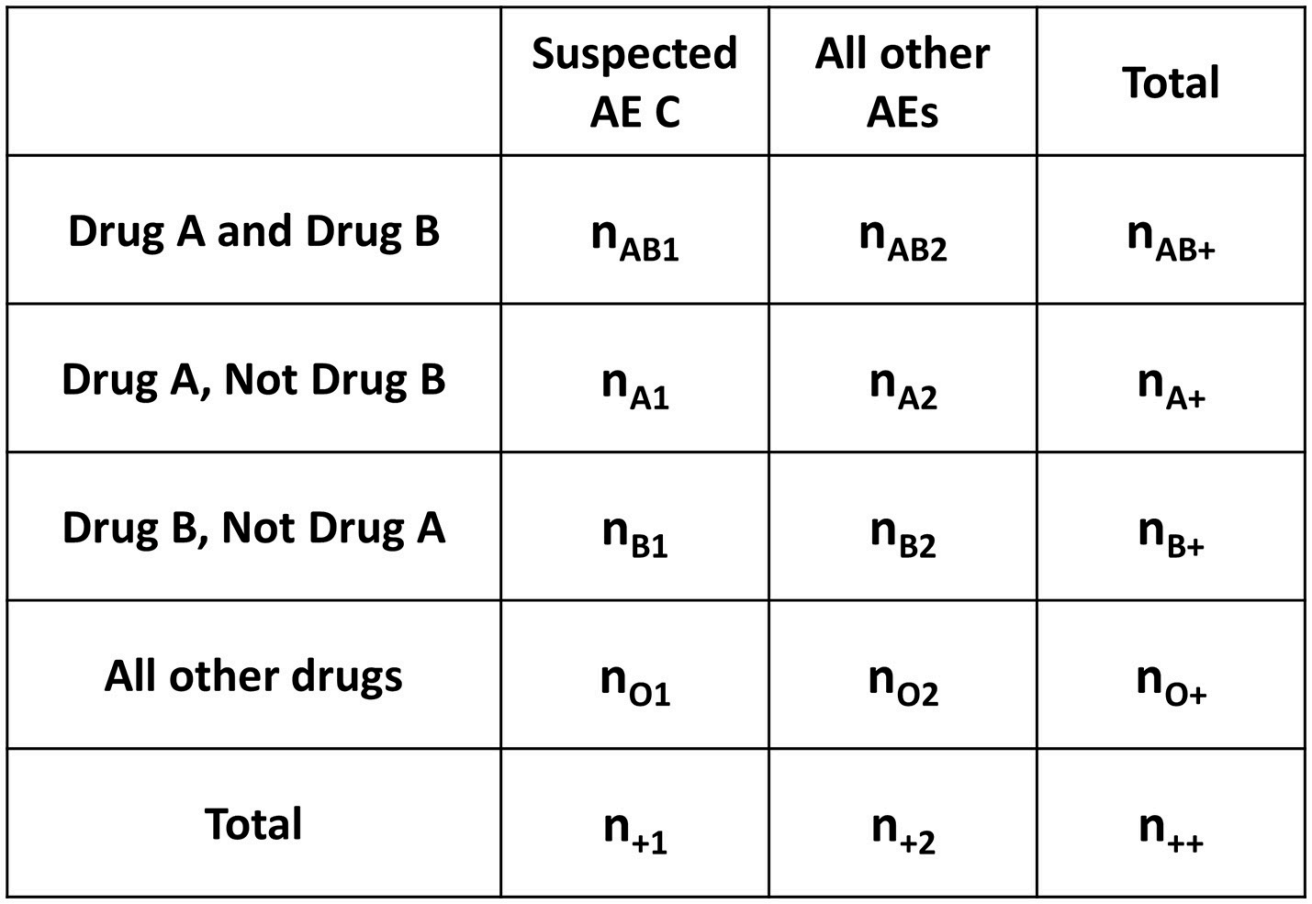
Four-by-two contingency table for AR from the Venn diagram in Figure 2. AR, association rule mining; AE, adverse events; n, the number of reports (e.g., n_++_, the number of all reports; n_AB1_, the number of Drug A and Drug B induced AE C reports).

In this paper, we propose AR as an alternative to the conventional ADE search methods for DDI. However, in this study, because the degree of the influence on Drug A-induced AE C by Drug B is used as an indicator of the DDI, the association rule “B → A ∩ C” (where B is Drug B and A ∩ C is the Drug A-induced AE C) was used instead of the typically used association rule “A ∩ B → C” (where A is Drug A, B is Drug B, and C is the AE C). In association rule “A ∩ B → C”, the signal by the combination of Drug A and Drug B is detected, but the influence due to the addition of Drug B cannot be investigated. In addition, we used “lift” and “conviction” as the detection criteria for searching the association rule. The calculation methods are shown in Figures 2, 3 and the following formulas.

$$\begin{array}{l} \text{lift }\left(\text{B}\to \text{A }\cap \text{ C}\right)\\ =\text{confidence }\left(\text{B}\to \text{A }\cap \text{ C}\right)/\text{support }\left(\text{A }\cap \text{ C}\right)\\ =\left\{{\text{n}}_{\text{AB}1}/\left({\text{n}}_{\text{AB}+}+{\text{n}}_{\text{B}+}\right)\right\}/\left\{\left({\text{n}}_{\text{AB}1}+{\text{n}}_{\text{A}1}\right)/{\text{n}}_{++}\right\}\\ \text{conviction }\left(\text{B}\to \text{A}\cap \text{C}\right)\\ =\left(1-\text{Support }\left(\text{A }\cap \text{ C}\right)\right)/\left(1-\text{Confidence }\left(\text{B}\to \text{A }\cap \text{ C}\right)\right)\\ =\left\{1-\left({\text{n}}_{\text{AB}1}+{\text{n}}_{\text{A}1}\right)/{\text{n}}_{++}\right\}/\left\{1-{\text{n}}_{\text{AB}1}/\left({\text{n}}_{\text{AB}+}+{\text{n}}_{\text{B}+}\right)\right\} \end{array}$$

“Lift” is an index that indicates the relative magnitude of the probability of observing A ∩ C under the condition of B, compared to the overall probability of observing A ∩ C. When lift = 1, the two events, B and A ∩ C, are independent of each other. When the lift value is greater than one, the two events, B and A ∩ C, are not independent, and the higher the value, the greater the relevance of the interaction (Hahsler et al., 2005).

On the other hand, “conviction” is an indicator that evaluates whether the rule makes a wrong prediction, paying particular attention to the exclusion event of the conclusion part of the obtained rule. If the conviction value is large, it is less likely that the conclusion A ∩ C is not true for the premise B (Brin et al., 1997).

In this study, lift > 1 and conviction > 1 were used as the detection standard for the AR method. However, in order to investigate the power of detection, because the combination of drugs for which the risk index is detected by the CR as the target was assumed to be true data, it was found that n_AB1_ < 3 was not detected in the “combined risk ratio.” That combination was therefore excluded from the signal and n_AB1_ ≥ 3 was added to the detection condition.

AR Criteria of DDI for this study:

$$\begin{array}{l} {\text{n}}_{\text{AB}1}\;\geq \;3,\text{ Lift }\left(\text{B}\to \text{A }\cap \text{ C}\right)\;>\;1\text{ and Conviction }\left(\text{B}\to \text{A }\cap \text{ C}\right)\;>1 \end{array}$$

### Combination risk ratio model

Susuta et al. proposed a CR model to generate DDI signals. In their method, AE C as DDI risk of drug A and drug B is estimated by the following procedure (Susuta and Takahashi, 2014):

1. PRR, which is a statistical signal detection indicator, is used to evaluate the increase in AE C as DDI risk of Drug A and Drug B.
2. PRR about one drug (= Drug A) of two target suspicious drugs is calculated individually.
3. The case where the ratio of the PRR of the two drug concomitant usage to the PRR obtained individually exceeds two is judged as a DDI risk.

$$\begin{array}{l} \text{DDI risk ratio}=\text{PR}{\text{R}}_{\text{Drug A and B}}/\text{PR}{\text{R}}_{\text{Drug A}}\\ \text{DDI risk criteria}:{\text{n}}_{\text{AB}1}\;\geq \;3,\text{PR}{\text{R}}_{\text{Drug A and B}}>2\text{and }\chi^{2}{\;}_{\text{Drug A and B}}\\ >\;4\text{ and DDI risk ratio }>2 \end{array}$$

### Additive interaction model

Thakrar et al. proposed an AI model to generate DDI signals (Thakrar et al., 2007). In their proposed method, under the additive assumption, no interaction is established when the excess risk associated with Drug A in the absence of Drug B is the same as the excess risk associated with Drug A in the presence of Drug B:

$$\begin{array}{lll} \text{risk}{\;}_{\left(\text{Drug A},\text{ not B}\right)}-\text{risk}{\;}_{\left(\text{not Drug A},\text{ not B}\right)} & = & \text{risk}{\;}_{\left(\text{Drug A},\text{ B}\right)}\\ -\text{risk}{\;}_{\left(\text{not Drug A},\text{ B}\right)} \end{array}$$

This equality implies:

$$\begin{array}{l} \text{Risk Difference }{\left(\text{RD}\right)}_{11}=\left(\text{R}{\text{D}}_{10}+\text{R}{\text{D}}_{01}\right) \end{array}$$

i.e. the excess risk associated with the combination is the same as the sum of the excess risks associated with each exposure in the absence of the other, where RD_11_ = risk _(DrugA,B)_ – risk _(notDrugA,notB)_ is the excess risk associated with the two drugs in combination and similarly for RD_10_ and RD_01_.

**Figure 4:**
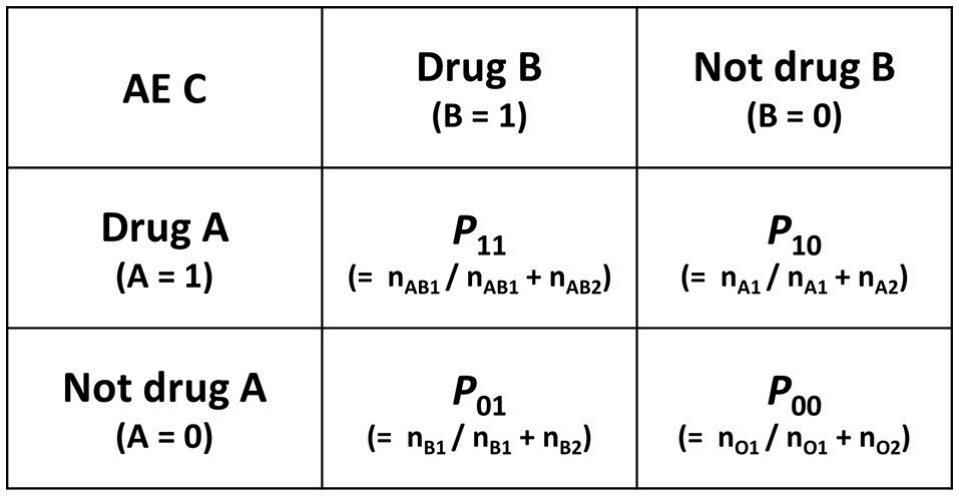
Two-by-two contingency table for calculation of AI and MI. AI, additive interaction; MI, multiplicative interaction; AE, adverse events; n, the number of reports (e.g., n_AB1_, the number of Drug A and Drug B induced AE C reports), P, the proporation of reports (e.g., P_11_,the proportion of Drug A and Drug B induced AE C report to Drug A and Drug B induced all AEs).

The AI is calculated from the two-by-two contingency table shown in Figure 4 and the following formulas.

$$\begin{array}{l} \text{AI}={\text{RD}}_{11}-({\text{RD}}_{10}+{\text{RD}}_{01})=(P_{11}-P_{00})-\{(P_{10}-P_{00})\\ \;+(P_{01}-P_{00})\}=P_{11}-P_{10}-P_{01}+P_{00} \end{array}$$

*P*: the proportion of reports

(e.g. *P*_11_: the proportion of Drug A and Drug B induced reports to Drug A and Dug B induced all AEs)

When RD_11_ > RD_10_ + RD_01_ (i.e., RD_11_ – (RD_10_ + RD_01_) > 0) there is a potential interaction with an increased risk for the combination compared with that expected based on the individual drugs. On the other hand, in the absence of an interaction under the additive assumption, the excess risk associated with the combination is the same as the sum of the excess risk associated with each drug separately (Thakrar et al., 2007). However, in this study, because it was found that n_AB1_ < 3 is not detected in the “combined risk ratio,” that combination was excluded from the signal, and n_AB1_ ≥ 3 was added to the detection condition.

### Multiplicative interaction model

Thakrar et al. proposed an MI model to generate DDI signals (Thakrar et al., 2007). In their proposed model, when there is no interaction on the multiplicative scale, the relative risk associated with Drug A is the same in both the absence and presence of exposure to Drug B.

$$\begin{array}{l} \text{ris}{\text{k}}_{\left(\text{Drug A},\text{ not B}\right)}/\text{ris}{\text{k}}_{\left(\text{not Drug A},\text{ not B}\right)}\\ =\text{ris}{\text{k}}_{\left(\text{Drug A},\text{ B}\right)}/\text{ris}{\text{k}}_{\left(\text{not Drug A},\text{ B}\right)} \end{array}$$

This equality implies:

$$\begin{array}{l} \text{Risk Ratio }{\left(\text{RR}\right)}_{11}=\left(\text{R}{\text{R}}_{10}\times \text{R}{\text{R}}_{01}\right) \end{array}$$

where RR_11_ = risk _(DrugA,B)_/risk _(notDrugA,notB)_ is the relative risk associated with the two drugs in combination and similarly for RR_10_ and RR_01_.

The MI is calculated from the two-by-two contingency table shown in Figure 4 and the following formulas.

$$\begin{array}{lll} \text{MI} & = & \text{R}{\text{R}}_{11}/\left(\text{R}{\text{R}}_{10}\times \text{R}{\text{R}}_{01}\right)=\left(P_{11}/P_{00}\right)/\left\{\left(P_{10}/P_{00}\right)\times \left(P_{01}/P_{00}\right)\right\}\\ = & \left(P_{11}\times P_{00}\right)/\left(P_{10}\times P_{01}\right) \end{array}$$

If RR_11_/(RR_10_ × RR_01_) is statistically different from one, it is considered evidence of an interaction. In particular, whenever this measure is greater than one, there is a positive interaction that is of interest from a safety perspective. In such a situation, the relative risk associated with two drugs administered in combination is greater than the product of the relative risks associated with each drug separately (Thakrar et al., 2007). However, in this current study, because it was found that n < 3 is not detected in the “combined risk ratio,” that combination was excluded from the signal, and n ≥ 3 was added to the detection condition.

### Application of the models

Since true data of unknown DDIs cannot be prepared, in this study, we treated the drug-drug combination detected CR model as true data. This model is used for searching DDI signals in Japan (Noguchi et al., 2015; Mizuno et al., 2016), but, if a calculation model that simply creates combinations from the database was used, the number of combinations that need to be calculated would be enormous. Therefore, there is a need for a simple method to obtain a search result similar to this method.

The accuracy of signal detection using the AR, AI, and MI methods was examined using sensitivity, specificity, Youden's index, positive predictive value (PPV), negative predictive value (NPV), *F*-score and area under the receiver operating characteristic (ROC) curve (AUC).

### Analysis software

Data management and analyses were performed using Visual Mining Studio software (version 8.1; Mathematical Systems, Inc. Tokyo, Japan). Drawing of the ROC curve and AUC calculation were performed using JMP 11.2.0 (SAS Institute Inc.,).

## Results

Among all the cases analyzed (374,327 cases), there were 7,848 drug–AE (SJS) combinations. The number of signals detected using the “combined risk ratio” was 1,672 pairs.

Table 1 shows the signal detection power of AR, AI, and MI. For AR, the sensitivity was 99.05%, specificity was 92.60%, Youden's index was 0.917, positive predictive value (PPV) was 78.57%, negative predictive value (NPV) was 99.72% and *F*-score was 0.876. For AI, the sensitivity was 95.62%, specificity was 96.92%, Youden's index was 0.925, PPV was 89.47%, NPV was 98.78% and *F*-score was 0.924. For MI, the sensitivity was 65.46%, specificity was 98.78%, Youden's index was 0.642, PPV was 93.64%, NPV was 91.26% and *F*-score was 0.771. Among the three models, AR had the highest sensitivity and the highest NPV, whereas MI had the highest Specificity and the highest PPV. On the other hand, for Youden's index and *F*-score, AI had slightly higher values than those obtained by the AR model proposed in this study.

**Table 1:** Ability of AR, AI, and MI to detect signals.

**Models**	**Sensitivity (%)**	**Specificity (%)**	**Youden's index**	**PPV (%)**	**NPV (%)**	**F-score**
AR	99.05	92.60	0.917	78.57	99.72	0.876
AI	95.62	96.92	0.925	89.47	98.78	0.924
MI	65.46	98.78	0.642	93.64	91.26	0.771

*AR, Association rule mining; AI, additive interaction model (= risk difference), MI, multiplicative interaction model (= risk ratio), PPV, positive predictive value; NPV, negative predictive value*.

**Figure 5:**
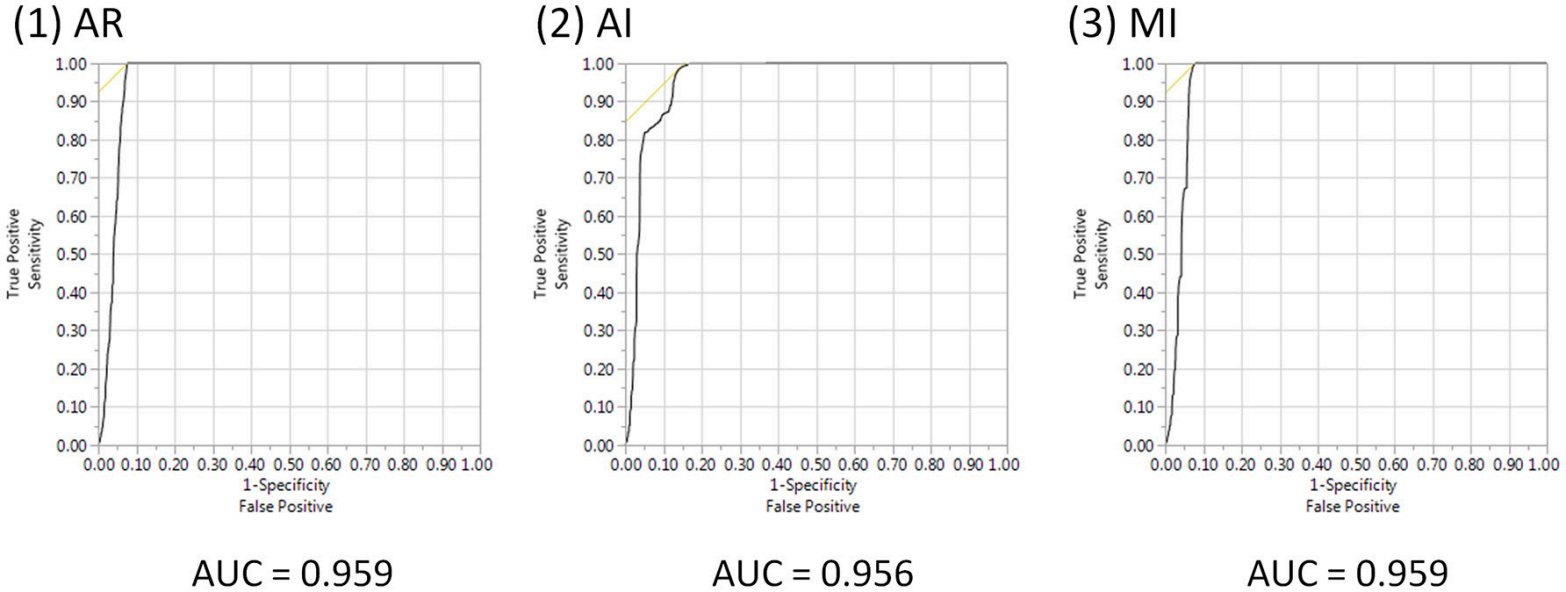
ROC curve and AUCs of AR, AI, and MI. AUE, area under the ROC curve; AR, association rule mining; AI, additive interaction; MI, multiplicative interaction.

Figure 5 shows the ROC curve. For AR, the AUC was 0.959; for AI, the AUC was 0.956; and for MI, the AUC was 0.959. For all three models, the AUC was greater than 0.95, which shows that AR has detection power that is on par with conventional methods such as AI and MI.

**Figure 6:**
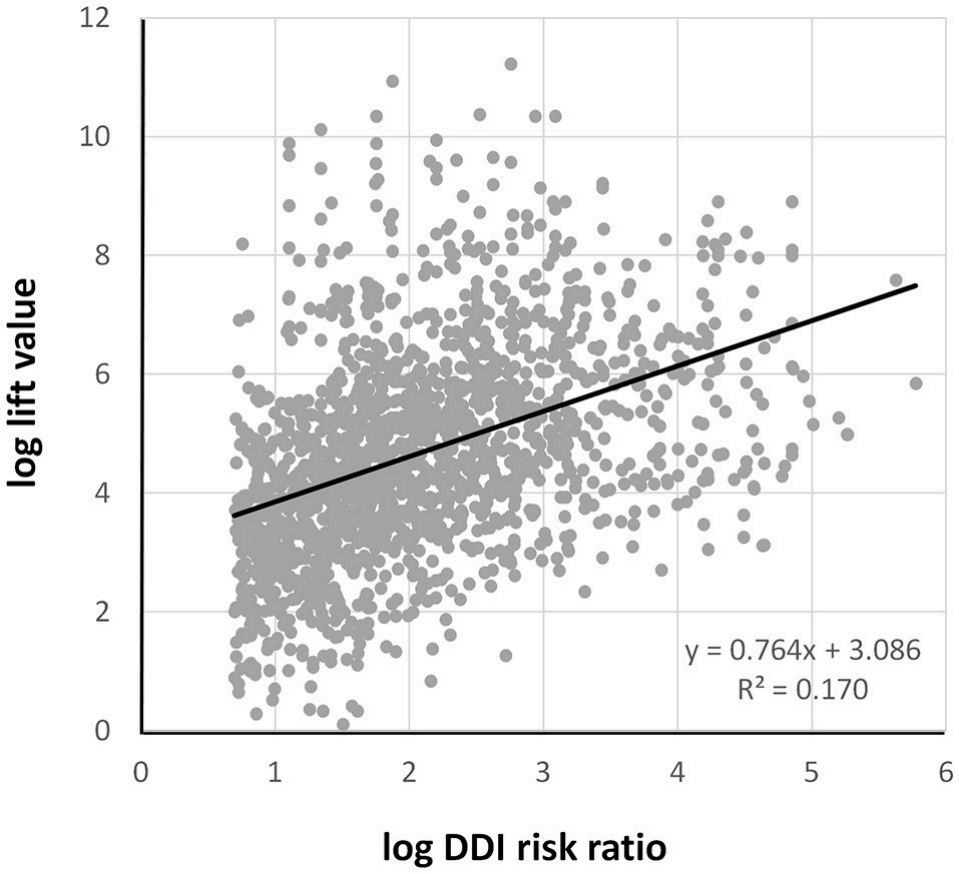
The correlation between log lift value and log DDI risk ratio. DDI, drug-drug interaction.

Figure 6 shows the correlation between the lift value and DDI risk ratio signal intensity. These show a positive correlation, the decision coefficient (R2) = 0.170.

Since there are many combinations of SJS-expressing drugs with 7848 pairs, in this paper, we will explain the case where Drug B is a combination that is loxoprofen. There were 195 pairs where Drug B was loxoprofen. Among them, 67 pairs of signals were detected. Table 2 shows some of them.

**Table 2:** Signal of some combination of drug B: loxoprofen as examples of DDI.

**Drug A**	**n_*AB*_**	**Combination risk ratio**			**Association rule mining**	
		**PRR _Drug A and B_**	**χ^2^_Drug A and B_**	**DDI risk ratio**	**lift**	**conviction**
Carbamazepine	20	18.70	320.29	2.75	4.77	1.00 (1.00343)
Clarithromycin	34	16.52	483.04	2.81	14.28	1.00 (1.00069)
lamotrigine	3	23.76	45.17	1.79	0.66	1.00 (0.99966)
phenytoin	1	9.90	1.59	1.35	0.89	1.00 (0.99997)

*DDI, drug-drug interaction; n_AB_, the number of Drug A and Drug B induced AE C reports; PRR, proportional reporting ratio*.

## Discussion

Whereas previous reports on the search for drug-induced AEs using SRS are numerous, reports on drug interactions are limited. This is because in methods that use a safety signal indicator, which is frequently used in pharmacovigilance, a huge number of combinations must be prepared when signal detection is performed, and each risk index must be calculated, which makes interaction search appear unrealistic.

A potential solution to this issue would be to utilize AR. However, although AR is often used to efficiently analyze large datasets, there are only a few examples of it being used in the medical field, especially in SRS analysis.

Wang et al. recently conducted a simulation study that is considered the gold standard of AR signal detection criteria for searching for ADEs due to single drugs (Wang et al., 2012). However, there are few examples of utilization of AR for early detection of drug interaction.

The method proposed by Susuta et al. can be considered as a prior research method in the search for DDI induced ADEs (Susuta and Takahashi, 2014). In this study, we treated the drug-drug combination detected by Susuta et al.'s method (= CR) as true data. Then, we evaluated the detection power of our proposed method by calculating the sensitivity, specificity, PPV, NPV, and *F*-score.

In general, when searching for AE C with drugs A and B, the AR model uses “A ∩ B → C”. However, this model only shows the association between the combination used and the AE C, it does not show the influence of DDI. To show the influence of DDI, the addition of Drug B requires that the indicator be larger than the original Drug A -induced AE C (“B → A ∩ C”). Therefore, in this study, the degree of influence on Drug A-induced AE C by Drug B is used as an indicator of the DDI. In this way, the lift value calculated in this study is a variation value of the probability of occurrence of AE C caused by Drug A by using Drug B in combination.

For AR Criteria, the lift value is common. However, an indicator that evaluates whether the rule has made a wrong prediction needs to be included among the criteria. Therefore, the conviction value was also added as a criterion.

The sensitivity for AR was 99.05%. That is, using AR, it was revealed that almost all of the combination of drug interactions expressing SJS indicated as signal detection in the combination risk index was detected. In addition, the sensitivity of AR was higher than both the 95.62% of AI and the 65.46% of MI, which are conventional methods. For Youden's index, which considers sensitivity to sensitivity, AR was 0.917, which is comparable to the 0.925 of AI, and exceeds the 0.642 of MI. Also, for *F*-score, AR was 0.876, which is near to the 0.924 of AI, and exceeds the 0.771 of MI. From these results, it is clear that the detection accuracy of AR is as high as that of AI and higher than that of MI.

The NPV of AR was 99.72%, that of AI was 98.78%, and that of MI was 91.26%. None of the detection methods had a negative misdetection signal. Of the three methods, AR was the least erroneous. On the other hand, with PPV, AR: 78.57% was lower than AI: 89.47%, and MI: 93.64%.

This result is because data using positive combination risk index, which is one of the signal detection criteria of existing drug interaction, were used as true data. AR can detect a combination of DDI induced SJS that cannot be detected with a combination risk index; hence, there is a possibility that PPV was underestimated.

However, as true data of “true risk” containing “unknown AEs” does not exist in the world, these results for AR, AI, and MI cannot be verified. It merely showed combinations of drugs with possible risks due to combination of sensitivity index and high sensitivity for the CR proposed by Susuta et al.

Since AR model proposed by us in this paper is represented by this formula: “B → A ∩ C”, if “Drug B → AE C” is a known effect, “Drug B → Drug A ∩ AE C” might be considered to be true for a random Drug A. However, when we performed investigations in accordance with this suggestion, we encountered a case in which the result was not necessarily the same as this suggestion.

For example, When Drug B is loxoprofen, AE C is SJS, the lift value of “loxoprofen → SJS” is 5.78. As shown in Table 2, among some of the combinations where drug B is loxoprofen, DDI risk is detected when Drug A is carbamazepine or clarithromycin. On the other hand, when Drug A is lamotrigine, the lift value is 0.66. Also, when Drug A is phenytoin, the lift value is 0.89. Even if “Drug B → AE C” is well-known, “Drug B → random Drug A n AE C” is not always true.

The magnitude of the lift value and that of the DDI risk ratio signal intensity are positively correlated. However, the hypothesis obtained from the signal should be verified separately for ADEs with a large lift value.

The CR is used for searching DDI signals in Japan (Noguchi et al., 2015; Mizuno et al., 2016), and there is a need for a simple method to obtain a search result similar to this method. If a similar calculation method that simply creates combinations from the database was used instead of AR, the number of combinations would be enormous and it would be difficult to perform the calculations within a realistic time. However, in the AR method, the “Apriori algorithm” can be used to reduce the number of calculations. The Apriori algorithm is based on the principle that “support of a certain item set is always less than or equal to support of its partial item set” (Agrawal and Srikant, 1994). Therefore, it is not necessary to calculate indices for all combinations, as in the conventional methods including the CR.

In this study, to compare the signal detection powers, all combinations were calculated in the AR analysis without using the apriori algorithm. Therefore, it is unknown how much time can be shortened by the apriori algorithm, compared with the conventional method. However, the pairs that can be a combination of drug interactions are predicted to be enormous. In our proposed method, since the calculation can be simplified by apriori algorithm in practice, it is expected that the signal search time will definitely be shortened in actual search.

So, it is possible to efficiently perform a cross-sectional search aiming at early detection of drug interactions. Furthermore, our proposed method can be applied not only to JADER but also to the SRS of other countries, such as the US FDA Adverse Event Reporting System (FAERS).

In this study, the true data for verification was created by CR, not the “real” true data. Therefore, we cannot deny the possibility that false positives are detected. We could not verify all combinations. Therefore, our proposed method requires verification for ADEs other than SJS. However, although only a part of the large data is used, this result obtained in this study would suggest that calculation is easy with the same detection power as the conventional method.

Because SRS is the result of spontaneous reporting and is influenced by reporting bias including underreporting, the value of the signal easily varies depending on the time of investigation; therefore, the analysis result is not a true risk but a risk hypothesis. Consequently, in order to clarify the true risk, further fundamental pharmacological research and clinical research are needed based on the obtained risk hypothesis. However, in order to discover an unknown ADE at an early stage, the issue of how to establish this hypothesis early can be considered an important factor. In pharmacovigilance, building a correct hypothesis is also important, but we believe that simple methods such as our proposed method with the same detection power as the conventional methods are required.

## Availability of data and material

The Japanese Adverse Drug Event Report database (JADER). [http://www.info.pmda.go.jp/fukusayoudb/CsvDownload.jsp] (in Japanese only).

## Author contributions

Conceived and designed the experiments: YN and HT. Performed the experiments: YN, AU, MO, and HK. Analyzed the data: YN, IS, YK, AY, and HE. Contributed reagents, materials, analysis tools: YN, HT, and TT. Wrote the paper: YN and HT.

### Conflict of interest statement

The authors declare that the research was conducted in the absence of any commercial or financial relationships that could be construed as a potential conflict of interest.
